# Sources and Coverage of Medical News on Front Pages of US Newspapers

**DOI:** 10.1371/journal.pone.0006856

**Published:** 2009-09-02

**Authors:** William Y. Y. Lai, Trevor Lane, Alison Jones

**Affiliations:** 1 Journalism and Media Studies Centre, The University of Hong Kong, Pokfulam, Hong Kong, People's Republic of China; 2 Faculty of Dentistry, The University of Hong Kong, Prince Philip Dental Hospital, Hong Kong, People's Republic of China; 3 School of Medicine and Public Health, Faculty of Health, University of Newcastle, Newcastle, New South Wales, Australia; Science Commons, United States of America

## Abstract

**Background:**

Medical news that appears on newspaper front pages is intended to reach a wide audience, but how this type of medical news is prepared and distributed has not been systematically researched. We thus quantified the level of visibility achieved by front-page medical stories in the United States and analyzed their news sources.

**Methodology:**

Using the online resource *Newseum,* we investigated front-page newspaper coverage of four prominent medical stories, and a high-profile non-medical news story as a control, reported in the US in 2007. Two characteristics were quantified by two raters: which newspaper titles carried each target front-page story (interrater agreement, >96%; kappa, >0.92) and the news sources of each target story (interrater agreement, >94%; kappa, >0.91). National rankings of the top 200 US newspapers by audited circulation were used to quantify the extent of coverage as the proportion of the total circulation of ranked newspapers in *Newseum*.

**Findings:**

In total, 1630 front pages were searched. Each medical story appeared on the front pages of 85 to 117 (67.5%–78.7%) ranked newspaper titles that had a cumulative daily circulation of 23.1 to 33.4 million, or 61.8% to 88.4% of all newspapers. In contrast, the non-medical story achieved front-page coverage in 152 (99.3%) newspaper titles with a total circulation of 41.0 million, or 99.8% of all newspapers. Front-page medical stories varied in their sources, but the *Washington Post, Los Angeles Times, New York Times* and the *Associated Press* together supplied 61.7% of the total coverage of target front-page medical stories.

**Conclusion:**

Front-page coverage of medical news from different sources is more accurately revealed by analysis of circulation counts rather than of newspaper titles. Journals wishing to widen knowledge of research news and organizations with important health announcements should target at least the four dominant media organizations identified in this study.

## Introduction

Medical news coverage in newspapers plays an important role for both the public and medical professionals [1]–[9]. It is likely that the prominence editors give to different news stories is influenced by “newsworthiness” and news source, and is reflected by page allocation, which in turn affects readers' perceptions. For example, medical news that appears on newspapers' front pages is intended to reach a wide audience and gain maximum or immediate attention. Researchers have acknowledged the importance of front-page positioning of medical news[6], [9]–[12], and the Project for Excellence in Journalism has also explained the value of researching front-page stories over inside-page stories [13].

The characteristics of front-page medical news have not yet been systematically researched, probably because of the large workload involved in exhaustively searching all newspaper front pages. A practical approach would be to limit the analysis to particular medical topics or stories during a selected period in one country. But even previous studies evaluating newspaper coverage of certain medical topics in the US used limited and variable samples of newspapers ranging from the five highest-circulation newspapers to 36 high-circulation national and regional US newspapers [14]–[18]. An objective sampling method does not seem to exist yet for newspaper analyses. In addition, analysis of newspaper titles alone does not reflect audience reach.

This study thus used an online US newspaper resource and a national newspaper audit to quantify the extent of coverage, in terms of newspaper titles and total newspaper circulation, of selected front-page medical stories and to assess if a story's visibility is associated with its news source. We also investigated whether findings differed between high-profile medical and non-medical stories, and between high- and low-profile medical stories.

## Methods

### Data Collection

The data source was the *Newseum* (www.newseum.org), an online daily repository containing electronic front pages of more than 300 US newspapers. For 6 weeks in 2007 (October 12 to November 22), we collected front-page newspaper coverage of high-profile medical stories in the US. We first relied on *Newseum* editors' daily analyses to identify each day's 10 most interesting front pages and then confirmed that any medical stories had national or international relevance, and immediate or potential public health implications. The full selection of front pages is available daily at 08:30 hours (US Standard Eastern Time) and *Newseum* editors' analysis appears soon after. Because *Newseum* displays newspaper front pages for only 24 hours and archives only front pages of historical significance, we checked the site daily at 23:00 hours Hong Kong time (11:00 hours US Standard Eastern Time) and collected data during the specific days of interest.

Four different high-profile medical stories were reported during our search period. Story 1 appeared on Friday, October 12, 2007, and originated from a public health announcement made by a trade organization representing manufacturers of over-the-counter drugs that recommended the voluntary withdrawal of over-the-counter infant cold and cough medications [19]. Story 2, reported on Monday, October 15, 2007, originated from an annual national report on cancer in the US published in the journal *Cancer* [20]. Story 3, dated Wednesday, October 17, 2007, reported research findings showing an increase in the number of methicillin-resistant *Staphylococcus aureus* (MRSA) cases in the US and was based on an article published in the *Journal of the American Medical Association* [21]. Story 4, dated Wednesday, November 21, 2007, covered research reported in *Cell* [*22*] and *Science* [23] that described the generation of stem cells from skin cells.

A high-profile non-medical story was used as a positive control to determine the “maximum” level of front-page coverage. We chose the news story (Story 5) about gun shootings at the Virginia Polytechnic Institute and State University (Virginia Tech) in Blacksburg, Virginia, which was reported nationwide on Tuesday, April 17, 2007, because it was the highest-ranking solitary news event of 2007 according to *Time Magazine* [24] and newspaper front pages on that date had been archived by the *Newseum*. In that incident, a student killed 32 students and staff, and then himself, on the morning of April 16, 2007.

Newspaper titles on the chosen days were categorized by whether they covered the target medical or non-medical story on their front pages and by the source of the report. We first counted any mentions of the story topics, including banner headings and boxed or unboxed summaries, to measure total front-page coverage given by all newspaper titles. Next, author bylines were used to categorize the news sources as newspaper staff writers, news syndicates, or wire services. A news syndicate was defined as a newspaper publisher or publishing group producing media reports that are also simultaneously licensed to subscribers (e.g. *Washington Post Company, McClatchy Newspapers*). Wire services were defined as organizations that do not print or publish news but supply news to subscribers (e.g. *Associated Press*). If a staff writer had declared that the primary source was a syndicate or wire, we still classified the source as a staff writer. None of the summaries had bylines, whereas all full reports did. Any mentions of the target stories without a byline were thus classified as unauthored briefs and were excluded before odds ratios were calculated for each known source of each medical story, with Story 5 acting as the reference. We also calculated the proportion of unauthored briefs that were standalone or referred the reader to an inside page.

To determine the total daily circulation of all titles bearing each target story (i.e. front-page coverage by circulation), we used the March 31, 2007 quarterly edition of the Audit Bureau of Circulations report, *Top 200 Newspapers by Largest Reported Circulation* [*25*]. The Top 200 US newspapers, ranked according to their individual audited circulations, together have a cumulative daily circulation of 46.3 million. Accordingly, this part of the analysis was limited to the ranked newspapers that appeared in the *Newseum* during the specific days of interest. We therefore used three denominators in our analyses: 1) all newspaper titles; 2) ranked newspaper titles; and 3) total circulation of ranked newspapers.

### Other Medical News Reported on Front Pages

To further understand how newspapers prioritize medical stories, we identified all other front-page medical news reported on the same days as the four medical stories (labeled as low profile). We noted their topic and assessed their news sources and coverage by newspaper title, ranked title, and circulation, as described above.

### Coding and Statistical Analysis

Two trained coders used a coding form to review downloaded newspaper front pages. The two coding items were (a) whether the front page contained the target news story, and (b) whether the front-page story was produced by a newspaper staff writer, news syndicate, or wire service, or if it was an unauthored brief. The interrater agreement was high for deciding whether or not the five target news stories appeared on a front page (96.0%, 98.2%, 98.8%, 98.8%, 100.0% observed agreement; kappa = 0.92, 0.96, 0.98, 0.98, 1.00 for stories 1, 2, 3, 4, and 5, respectively), and for identifying the sources (94.8%, 97.0%, 97.6%, 95.8%, 94.4% observed agreement; kappa = 0.92, 0.94, 0.96, 0.94, 0.91 for stories 1, 2, 3, 4, and 5, respectively).

Interrater reliability (kappa) was calculated with SPSS version 15.0 (SPSS Inc, Chicago, Illinois), and chi-square analysis of categorical data was performed with JMP version 5.1 (SAS Institute, Cary, North Carolina). A P value of <0.05 was considered statistically significant; for multiple combinations (n = 10) of pairwise comparisons, a more stringent P value of <0.005 was used. Odds ratios and 95% CIs with reference to Story 5 were calculated using online software (www.hutchon.net/ConfidOR.htm). In chi-square comparisons of Top 200 newspapers, circulation figures were expressed as count per 100,000 to ensure comparable cell sizes.

## Results

### Media Characteristics Based on Newspaper Titles

In total, 1630 US newspaper front pages from *Newseum* were searched: 326, 328, 340, and 330 for medical stories of October 12, 15, 17, and November 21, 2007, respectively, and 306 for the control story of April 17, 2007. The proportions of newspaper titles carrying any front-page mention (total front-page coverage by title) were 63.5%, 44.5%, 56.2%, 61.8%, and 99.0% for Stories 1 to 5, respectively (Table 1). Wire services were the most frequently used news source for all target front-page stories.

**Table 1:** Coverage and Sources of Selected High-profile Front-page Stories in US Newspapers in *Newseum*, by Newspaper Title.

	Story 1#		Story 2†		Story 3 ^		Story 4Δ		Story 5 (Control)‡
Date	Oct 12, 2007		Oct 15, 2007		Oct 17, 2007		Nov 21, 2007		Apr 17, 2007
	*No. (%)* *	Odds ratio (95% CI)	*No. (%)* *	Odds ratio (95% CI)	*No. (%)* *	Odds ratio (95% CI)	*No. (%)* *	Odds ratio (95% CI)	*No. (%)* *
**All newspaper titles**	**326 (100)**	**-**	**328 (100)**	**-**	**340 (100)**	**-**	**330 (100)**	**-**	**306 (100)**
Total coverage	207 (63.5)	-	146 (44.5)	-	191 (56.2)	-	204 (61.8)	-	303 (99.0)
News source (% of coverage):									
* Staff writers*	29 (14.0)	-	4 (2.7)	-	17 (8.9)	-	22 (10.8)	-	37 (12.2)
* News syndicates*	30 (14.5)	-	22 (15.1)	-	56 (29.3)	-	63 (30.9)	-	71 (23.4)
* Wire services*	76 (36.7)	-	90 (61.6)	-	64 (33.5)	-	76 (37.3)	-	164 (54.1)
* Unauthored briefs* ∇	72 (34.8)	-	30 (20.5)	-	54 (28.3)	-	43 (21.1)	-	31 (10.2)
Coverage, excluding unauthored briefs	135 (41.4)	***0.466 (0.357 to 0.608)***	116 (35.4)	***0.398 (0.305 to 0.520)***	137 (40.3)	***0.453 (0.351 to 0.586)***	161 (48.8)	***0.549 (0.428 to 0.704)***	272 (88.9)
News source (% of coverage):									
* Staff writers*	29 (21.5)	1.579 (0.931 to 2.678)	4 (3.4)	***0.253 (0.075 to 0.766)***	17 (12.4)	0.912 (0.496 to 1.679)	22 (13.7)	1.005 (0.572 to 1.76)	37 (13.6)
* News syndicates*	30 (22.2)	0.851 (0.530 to 1.368)	22 (19.0)	0.727 (0.430 to 1.229)	56 (40.9)	***1.566 (1.043 to 2.350)***	63 (39.1)	***1.499 (1.014 to 2.217)***	71 (26.1)
* Wire services*	76 (56.3)	0.934 (0.664 to 1.314)	90 (77.6)	1.287 (0.919 to 1.802)	64 (46.7)	0.775 (0.544 to 1.104)	76 (47.2)	0.783 (0.560 to 1.09)	164 (60.3)

# Cough and cold medicines withdrawn from retail outlets.

† US cancer death rates drop.

^ Increase in methicillin-resistant *Staphylococcus aureus* cases.

Δ Skins cells transformed into stem cells.

‡ Virginia Polytechnic Institute and State University (Virginia Tech) shootings.

* Because of rounding, not all percentages total 100.

∇ *Small box briefs, banner headings and anonymous short summaries.*

Among the 126 to 156 Top 200 newspapers that appeared in *Newseum* (capture rate, 61.1%–75.7%; n = 206), the front pages of 85 to 117 titles carried any mention of the target medical stories, corresponding to a total front-page coverage ranging from 67.5% to 78.7% of ranked newspaper titles. In contrast, 152, or 99.3%, of ranked newspaper titles in *Newseum* carried any mention of the control story (Table 2). Of the unauthored briefs mentioning the four medical news topics, 81.9% (163/199) were boxed announcements or banner heads and the remainder were anonymous short summaries. Nearly all (98%; 195/199) unauthored briefs referred the reader to a full story on an inside page.

**Table 2:** Coverage and Sources of Selected High-profile Front-page Stories in US Newspapers in *Newseum*, by Ranked and Unranked Newspaper Title.

	Story 1#		Story 2†		Story 3 ^		Story 4Δ		Story 5 (control)‡
	*No. (%)* *	Odds ratio (95% CI)	*No. (%)* *	Odds ratio (95% CI)	*No. (%)* *	Odds ratio (95% CI)	*No. (%)* *	Odds ratio (95% CI)	*No. (%)* *
**Number of Top 200 titles in ** ***Newseum***	**138 (100)**	**-**	**126 (100)**	**-**	**156 (100)**	**-**	**136 (100)**	**-**	**153 (100)**
Total coverage in ranked title*s*	108 (78.3)	0.774 (0.538 to 1.113)	85 (67.5)	***0.679 (0.476 to 0.968)***	117 (75.0)	0.755 (0.544 to 1.048)	107 (78.7)	0.792 (0.565 to 1.111)	152 (99.3)
Coverage, excluding unauthored briefs, in ranked titles	83 (60.1)	***0.644 (0.451 to 0.918)***	66 (52.4)	***0.560 (0.385 to 0.815)***	89 (57.1)	***0.610 (0.432 to 0.863)***	97 (71.3)	0.763 (0.540 to 1.079)	143 (93.5)
News source (% of coverage):									
*Staff writers*	25 (30.1)	1.723 (0.930 to 3.193)	4 (6.1)	0.347 (0.116 to 1.036)	14 (15.7)	0.900 (0.444 to 1.822)	19 (19.6)	1.120 (0.585 to 2.146)	25 (17.5)
*News syndicates*	23 (27.7)	0.777 (0.443to 1.363)	17 (25.8)	0.722 (0.388 to 1.345)	42 (47.2)	1.323 (0.813 to 2.153)	41 (42.3)	1.185 (0.730 to 1.926)	51 (35.7)
*Wire services*	35 (42.2)	0.900 (0.551 to 1.469)	45 (68.2)	1.455 (0.903 to 2.345)	33 (37.1)	0.791 (0.483 to 1.297)	37 (38.1)	0.814 (0.505 to 1.312)	67 (46.9)
**Number of unranked titles in ** ***Newseum***	**188 (100)**		**202 (100)**		**184 (100)**		**194 (100)**		**153 (100)**
Coverage, excluding unauthored briefs, in unranked titles	52 (27.7)	***0.328 (0.223 to 0.483)***	50 (24.8)	***0.294 (0.199 to 0.433)***	48 (26.1)	***0.309 (0.209 to 0.459)***	65 (33.5)	***0.397 (0.276 to 0.573)***	129 (84.3)
News source (% of coverage):									
*Staff writers*	4 (7.7)	0.827 (0.255 to 2.682)	0 (0)	0	3 (6.3)	0.672 (0.182 to 2.485)	3 (4.6)	0.496 (0.135 to 1.820)	12 (9.3)
*News syndicates*	7 (13.5)	0.868 (0.346 to 2.177)	5 (10.0)	0.645 (0.230 to 1.812)	14 (29.2)	1.881 (0.881 to 4.019)	23 (35.4)	***2.282 (1.169 to 4.457)***	20 (15.5)
*Wire services*	41 (78.8)	1.049 (0.645 to 1.706)	45 (90.0)	1.197 (0.740 to 1.937)	31 (64.6)	0.859 (0.509 to 1.449)	39 (60.0)	0.798 (0.496 to 1.285)	97 (75.2)

# Cough and cold medicines withdrawn from retail outlets.

† US cancer death rates drop.

^ Increase in methicillin-resistant *Staphylococcus aureus* cases.

Δ Skins cells transformed into stem cells.

‡ Virginia Polytechnic Institute and State University (Virginia Tech) shootings.

* Because of rounding, not all percentages total 100.

#### Characteristics Among All Newspaper Titles

After unauthored briefs had been excluded, front-page coverage was significantly less likely for the medical stories than the non-medical story (odds ratios, 0.398 to 0.549; Table 1). Wire services remained the most common source for all stories. Story 1's profile of news sources was similar to that of Story 5. Story 2 was significantly less likely than Story 5 to be written by staff writers, and both Stories 3 and 4 were significantly more likely to be supplied by news syndicates.

Chi-square analyses showed that Stories 1, 3, and 4 were not significantly different from Story 5 in profiles of news sources (P = 0.1282, P = 0.0094, and P = 0.0136, respectively), whereas Story 2 was (P = 0.0005). Stories 2, 3, and 4 were significantly different from Story 1 (P<0.0001, P = 0.0023, P = 0.0049, respectively). Furthermore, Stories 3 and 4 were not significantly different from one another (P = 0.9279) but were each significantly different from Story 2 (P<0.0001 for both).

#### Characteristics Among Ranked Newspaper Titles

Among *Newseum* newspapers included in the Top 200 rankings, Stories 1, 2, and 3 were significantly less likely than Story 5 to appear on front pages (odds ratios, 0.560 to 0.644; Table 2), after unauthored briefs had been excluded. Stories 1, 2, and 5 most frequently used wire services as news sources, whereas Stories 3 and 4 most frequently used news syndicates; however, no significant differences in sources between the medical and non-medical stories were detected.

Chi-square analyses confirmed that Stories 1, 2, 3, and 4 were not significantly different from Story 5 in profiles of news sources (P = 0.0841, P = 0.0067, P = 0.2095, and P = 0.4032, respectively). Story 2 was significantly different from Story 1 (P = 0.0002) but Stories 3 and 4 were not (P = 0.0134 and P = 0.0855, respectively). Furthermore, Stories 3 and 4 were not significantly different from one another (P = 0.720) but were each significantly different from Story 2 (P = 0.0005 and P = 0.0004, respectively).

Among *Newseum* newspapers not appearing in the Top 200 rankings, the four medical stories were again significantly less likely than the non-medical one to appear on front pages (odds ratios, 0.294 to 0.397; Table 2). All five stories most commonly came from wire services, although Story 4 was significantly more likely than Story 5 to have been reported by news syndicates.

### Media Characteristics Based on Weighted Circulation

Analyses of *Newseum* newspapers included in the Top 200 rankings and circulation figures of the Top 200 newspapers revealed that each target medical story received any mention in a total of 23.1 to 33.4 million newspapers nationally, which corresponded to total front-page coverage rates, by circulation count, ranging from 61.8% to 88.4% of all newspapers. In contrast, the high-profile non-medical story received any mention in a total circulation of 41.0 million or 99.8% of all newspapers (Table 3). Unauthored briefs were now the most common news source for Stories 1, 2 and 3 (34.1%, 37.0%, and 33.6%, respectively), but least common for Stories 4 and 5 (7.2% and 8.0%, respectively); staff written reports were the most common source for Stories 4 and 5.

**Table 3:** Coverage and Sources of Selected High-profile Front-page Stories in Ranked US Newspapers in *Newseum*, by Total Circulation.

	Story 1#		Story 2†		Story 3 ^		Story 4Δ		Story 5 (control)‡
	*No. per 100,000 (%)* *	Odds ratio (95% CI)	*No. per 100,000 (%)* *	Odds ratio (95% CI)	*No. per 100,000 (%)* *	Odds ratio (95% CI)	*No. per 100,000 (%)* *	Odds ratio (95% CI)	*No. per 100,000 (%)* *
**Circulation of Top 200 titles in ** ***Newseum***	**385 (100)**		**374 (100)**		**402 (100)**		**378 (100)**		**411 (100)**
Total coverage	314 (81.6)	-	231 (61.8)	-	293 (72.9)	-	334 (88.4)	-	410 (99.8)
News source (% of coverage):									
*Staff writers*	103 (33.0)	-	32 (13.6)	-	49 (16.8)	-	138 (41.3)	-	147 (35.8)
*News syndicates*	51 (16.4)	-	44 (18.9)	-	93 (31.8)	-	110 (32.9)	-	108 (26.4)
*Wire services*	52 (16.5)	-	70 (30.4)	-	52 (17.8)	-	62 (18.6)	-	123 (29.9)
*Unauthored briefs* ∇	107 (34.1)	-	86 (37.0)	-	98 (33.6)	-	24 (7.2)	-	33 (8.0)
Coverage, excluding unauthored briefs	207 (53.8)	***0.586 (0.471 to 0.730)***	146 (39.0)	***0.426 (0.336 to 0.539)***	195 (48.5)	***0.529 (0.424 to 0.660)***	310 (82.0)	0.894 (0.728 to 1.098)	377 (91.7)
News source (% of coverage):									
*Staff writers*	103 (50.0)	1.276 (0.942 to 1.729)	32 (21.7)	***0.562 (0.367 to 0.862)***	49 (25.3)	***0.644 (0.446 to 0.93)***	138 (44.5)	1.142 (0.866 to 1.506)	147 (38.9)
*News syndicates*	51 (24.9)	0.86 (0.592 to 1.25)	44 (30.1)	1.052 (0.706 to 1.568)	93 (47.9)	***1.665 (1.201 to 2.308)***	110 (35.5)	1.239 (0.913 to 1.681)	108 (28.7)
*Wire services*	52 (25.1)	0.77 (0.534 to 1.11)	70 (48.3)	***1.47 (1.035 to 2.086)***	52 (26.8)	0.817 (0.566 to 1.18)	62 (20.0)	***0.613 (0.436 to 0.862)***	123 (32.5)

# Cough and cold medicines withdrawn from retail outlets.

† US cancer death rates drop.

^ Increase in methicillin-resistant *Staphylococcus aureus* cases.

Δ Skins cells transformed into stem cells.

‡ Virginia Polytechnic Institute and State University (Virginia Tech) shootings.

* Because of rounding, not all percentages total 100.

∇ *Small box briefs, banner headings and anonymous short summaries.*

After unauthored briefs had been excluded, Stories 1, 2, and 3 were significantly less likely than Story 5 to receive front-page coverage (odds ratios, 0.426 to 0.586; Table 3). The most common medical news source differed by story. Stories 1, 4, and 5 most frequently originated from staff writers, Story 2 from wire services, and Story 3 from news syndicates. Odds ratios revealed that Story 1 had a similar news source profile to that of Story 5, whereas Stories 2 and 3 were significantly less likely than Story 5 to be prepared by staff writers. Story 2 was also more likely to be provided by wire services, and Story 3 was more likely to be provided by news syndicates. Story 4 was significantly less likely than Story 5 to come from wire services.

Chi-square analyses confirmed that Story 1 was not significantly different from Story 5 in profiles of news sources (P = 0.0315) but Stories 2, 3, and 4 were (P = 0.0003, P<0.0001, and P = 0.0009, respectively). Stories 2 and 3 were significantly different from Story 1 (P<0.0001 for both) but Story 4 was not (P = 0.0301). Furthermore, Stories 3 and 4 were significantly different from one another (P<0.0001), and from Story 2 (P = 0.0002 and P<0.0001, respectively).

### Comparison of Newspaper Titles and Weighted Circulation for Ranked Newspapers

For *Newseum* newspapers included in the Top 200 rankings, the chi-square test showed that the profile of known news sources as proportions of newspaper titles (60.1%, 52.4%, 57.1%, 71.3%, 93.5% for stories 1 to 5; Table 2) was significantly different from the profile as proportions of circulation counts (53.8%, 39.0%, 48.5%, 82.0%, 91.7%; Table 3) for Stories 1, 2, 4, and 5 (P = 0.0037, P = 0.0029, P<0.0001, and P<0.0001, respectively). Only Story 3 showed no significant difference (P = 0.0942).

### Main News Source Providers

The data based on newspaper titles (Table 1) showed that, after exclusion of unauthored briefs, news syndicates and wire services were together the main sources of the target front-page stories: 78.5%, 96.6%, 87.6%, 86.3%, and 86.4% for Stories 1 to 5. A similar trend (P = 0.8390) was observed when the sample was limited to ranked newspapers in *Newseum* (69.9%, 94.0%, 84.3%, 80.4%, and 82.6%; Table 2). In contrast, on the basis of newspaper circulation counts (Table 3), the major sources of news coverage varied by story and were less clear-cut.

The majority of target front-page medical stories in ranked newspapers that were reported by news syndicates came from three newspapers: the *Washington Post*, *Los Angeles Times*, and *New York Times* (Table 4). These three syndicates together supplied 79.6% (23,448,589/29,473,055) of all circulated newspapers displaying syndicated medical news stories in our sample, compared with 58.2% (6,300,357/10,825,900) for Story 5. These three newspapers actually use staff writers and usually publish syndicated stories on their own front pages; hence, they also accounted for 24.7% (7,936,113/32,187,394) of the target medical stories written by staff writers and 25.4% (3,730,079/14,664,290) of staff-written reports covering Story 5. The *Associated Press* contributed 90.0% (21,285,196/23,658,409) of all wire reports of the target front-page medical news stories, and 87.2% (10,680,996/12,255,742) for Story 5. Thus, three major newspapers and the *Associated Press* supplied 61.7% (52,669,898/85,318,858) of the total coverage of target medical stories with known news sources.

**Table 4:** Coverage and Main News Sources of Selected High-profile Front-page Stories in Ranked US Newspapers in *Newseum*, by Total Circulation.

	Story 1	Story 2	Story 3	Story 4	Story 5
Coverage, excluding unauthored briefs, by total circulation (%)	20,653,288 (100)	14,552,042 (100)	19,453,859 (100)	30,989,218 (100)	37,745,932 (100)
**Main news source, No (%)**					
Staff writers	10,333,493 (50)	3,150,461 (21.6)	4,930,709 (25.3)	13,772,731 (44.4)	14,664,290 (38.8)
	*25 Top 200 newspapers (rank: 4–6, 10, 11, 14, 15, 20, 22, 23, 23, 26, 27, 29, 32, 43, 49, 67, 67, 74, 76, 82, 141, 159, 176)*	*4 Top 200 newspapers (rank: 1, 45, 47, 108)*	*14 Top 200 newspapers (rank: 4, 6, 11, 25, 27–29, 84, 115, 121, 124, 154, 167, 197)*	*19 Top 200 newspapers (rank: 1–6, 10, 11, 21, 25–28, 42, 47, 49, 79, 102, 112)*	*25 Top 200 newspapers (rank: 1–6, 14, 16, 18, 22, 23, 25, 27, 28, 32, 47, 49, 55, 68, 102, 112, 146, 173, 182, 195)*
News syndicates	5,134,993 (24.9)	4,373,284 (30.1)	9,314,031 (47.9)	10,980,296 (35.4)	10,825,900 (28.7)
	*23 Top 200 newspapers (rank: 9, 13, 35, 38, 40, 45, 52, 52, 54, 61, 62, 73, 79, 81, 84, 87, 88, 89, 100, 114, 120, 121, 134)*	*17 Top 200 newspapers (rank: 9, 22, 24, 26, 33, 37, 44, 50, 53, 56, 61, 79, 100, 116, 119, 129, 165)*	*42 Top 200 newspapers (rank: 10, 13, 17, 22, 23, 24, 31, 33, 37, 40, 44, 45, 48, 52, 53, 54, 59, 61, 62, 63, 73, 78, 79, 80, 81, 86, 87, 89, 90, 94, 97, 99, 100, 101, 109, 120, 131, 134, 136, 160, 162, 185)*	*41 Top 200 newspapers (rank: 9, 12–14, 18, 20, 23, 30, 31–33, 35, 36, 37, 40, 43–45, 48, 53–55, 59, 60–63, 72, 80, 81, 86, 89, 90, 92, 94, 100, 128, 160, 162, 164, 183)*	*51 Top 200 newspapers (rank: 12, 12, 20, 21, 24, 26, 29, 30, 31, 36, 37, 39, 40, 41, 43, 44, 45, 48, 53, 54, 59, 62, 67, 71, 72, 73, 80, 81, 84, 89, 90, 92, 94, 97, 100, 101, 104, 106, 107, 124, 126, 128, 131, 140, 152, 155, 159, 160, 165, 169, 183)*
	*Washington Post syndicate (16 newspapers with cumulative circulation 3,486,452); New York Times syndicate (3 newspapers with circ 809,178); Los Angeles Times syndicate (1 newspaper with circ 266,594); USA Today syndicate (1 newspaper with circ 226,807); Newsday syndicate (1 newspaper with circ 226,807); Philadelphia syndicate (1 newspaper with circ 119,155)*	*New York Times syndicate (9 newspapers with cumulative circulation 2,587,414); – McClatchy syndicate (8 newspapers with circ 1,785,870)*	*Washington Post syndicate (25 newspapers with cumulative circulation 5,412,506); New York Times syndicate (6 newspapers with circ 1,331,580); Los Angeles Times syndicate (5 newspapers with circ 1,246,838); Other syndicates (6 newspapers with circ 1,323,107)*	*Los Angeles Times syndicate (19 newspapers with cumulative circulation 4,404,528); New York Times syndicate (7 newspapers with circ 2,057,936); Washington Post syndicate (8 newspapers with circ 1,845,563); Other syndicates (7 newspapers with circ 2,329,837)*	*Washington Post syndicate (26 newspapers with cumulative circulation 5,364,978); McClatchy syndicate (6 newspapers with circ 1,122,467); Los Angeles Times syndicate (3 newspapers with circ 564,811); New York Times syndicate (3 newspapers with circ 370,586); USA Today syndicate (3 newspapers with circ 341,763); Other syndicates (10 newspapers with circ 3,061,295)*
Wire services	5,184,802 (25.1)	7,028,297 (48.3)	5,209,119 (26.8)	6,236,191 (20.1)	12,255,742 (32.5)
	*35 Top 200 newspapers (rank: 30, 33, 34, 46, 48, 50, 56, 59, 60, 63, 71, 72, 75, 86, 90, 93, 94, 102, 107, 119, 123, 124, 128, 129, 140, 143, 148, 152, 155, 162, 164, 170, 173, 179, 189)*	*45 Top 200 newspapers (rank: 17, 28, 29, 30, 31, 34, 35, 52, 57, 60, 70, 71, 73, 75, 78, 80, 84, 86, 89, 93, 94, 97, 101, 104, 107, 113, 114, 120, 124, 126, 128, 141, 145, 147, 153, 155, 162, 163, 167, 173, 179, 183, 185, 195, 198)*	*33 Top 200 newspapers (rank: 15, 23, 26, 35, 42, 50, 52, 55, 60, 68, 70, 82, 85, 93, 106, 108, 116, 118, 119, 126, 129, 137, 141, 143, 148, 152, 155, 165, 170, 177, 183, 189, 194)*	*37 Top 200 newspapers (rank: 15, 17, 29, 34, 41, 46, 50, 52, 52, 57, 65, 68, 71, 75, 76, 82, 85, 88, 99, 105, 107, 108, 113, 116, 118, 124, 135, 139, 141, 143, 148, 153, 156, 165, 179, 189, 198)*	*67 Top 200 newspapers (rank: 9, 10, 11, 13, 15, 17, 23, 33, 34, 35, 38, 42, 46, 50, 52, 52, 57, 60, 61, 63, 70, 74, 75, 76, 77, 79, 82, 83, 85, 86, 88, 99, 103, 105, 109, 113, 115, 116, 116, 118, 123, 130, 135, 136, 137, 139, 141, 142, 143, 147, 148, 153, 156, 161, 162, 164, 170, 171, 179, 180, 181, 185, 186, 189, 197, 198, 200)*
	*Associated Press (33 newspapers with cumulative circulation 4,877,413); Others (2 newspapers with circ 307,389)*	*Associated Press (44 newspapers with cumulative circulation 6,957,502); Other (1 newspaper with circ 70,795)*	*Associated Press (29 newspapers with cumulative circulation 4,027,376);Others (4 newspapers with circ 1,181,743)*	*Associated Press (34 newspapers with cumulative circulation 5,422,905); Others (3 newspapers with circ 813,286)*	*Associated Press (61 newspapers with cumulative circulation 10,680,996); Others (6 newspapers with circ 1,574,746)*

Note: Ranked newspapers are listed to show origin of total cumulative circulation figures. The main contributors for News Syndicates and Wire Services with related circulation figures are also listed.

### Other Medical News Reported on Front Pages

On the same day as each of the target medical stories, 4.6% to 11.0% of newspapers carried a total of 5 to 17 additional or alternative front-page medical stories (Table 5). These lower-profile medical stories were mostly written by staff writers (50.0%–60.0%). On the basis of Top 200 newspaper circulation counts, the mean circulation of the stories appearing on the same day as Stories 1, 2, 3, and 4 was 150,000, 390,000, 230,000, and 300,000, respectively. The highest visibility achieved by a single low-profile medical story was coverage in 7 newspaper titles with a cumulative circulation of 0.9 million. This story, reported on the same day as Story 2, was based on a predictive test for Alzheimer disease that had been published in *Nature Medicine* [26]. Statistical comparisons between Stories 1 to 4 and these lower-profile medical stories were not performed because of small cell sizes.

**Table 5:** Coverage and Sources of Low-profile Front-page Medical Stories in US Newspapers in *Newseum*, by Newspaper Title and Total Circulation.

	Oct 12, 2007	Oct 15, 2007	Oct 17, 2007	Nov 21, 2007
*Number (percent)* *				
All newspaper titles	326 (100)	328 (100)	340 (100)	330 (100)
Total coverage	15 (4.6)	36 (11.0)	11 (3.2)	5 (1.5)
News source (% of coverage):				
* Staff writers*	*9 (60.0)*	*18 (50.0)*	*6 (54.5)*	*3 (60.0)*
* News syndicates*	*1 (6.7)*	*4 (11.1)*	*3 (27.3)*	*2 (40.0)*
* Wire services*	*2 (13.3)*	*10 (27.8)*	*1 (9.1)*	*0 (0)*
* Unauthored briefs* ∇	*3 (20.0)*	*4 (11.1)*	*1 (9.1)*	*0 (0)*
*[Circulation per 100,000]*				
Cumulative circulation in Top 200 titles	*[14.8]*	*[66.8]*	*[18.2]*	*[14.8]*
Number of different stories	*10* #	*17* †	*8* ^	*5* Δ
Mean cumulative daily circulation	*[1.5]*	*[3.9]*	*[2.3]*	*[3.0]*

* Because of rounding, not all percentages total 100.

∇ *Small box briefs, banner headings and anonymous short summaries.*

# Topics: Abortion (3), Food recall(3), Bacteria and chocolate (2), Kaposi's sarcoma, Space bacteria, Odor, Lead, Salmonella, Alcohol, Avian flu.

† Topics: Alzheimer Disease (7) [26], Diets (6), Autism (5), Medtronic device (4), Hypertension (2), Kidney swap, Hospital testing, Baby deaths, CPR intervention, Flu vaccine, Drug side effects, Genetics and diet, Depression, Sickle cell, Exercise, Obesity, Computer healthcare.

^ Topics: Physical examinations (3), Alcohol (2), Diet, Flu vaccine, Autism, Spina bifida, Heart, Caesarean birth rates.

Δ Topics: Health tips, Toxic vapors, *Staphylococcus* infection, AIDS, Illegal health devices.

## Discussion

In this study, we identified four high-profile front-page medical stories in US newspapers and quantified their media characteristics in terms of news source and coverage by title and total circulation. Each high-profile medical story received any front-page coverage in 67.5% to 78.7% of ranked newspaper titles in the *Newseum* repository and had a cumulative circulation ranging from 61.8% to 88.4% of ranked newspapers. In comparison, the “maximum” total front-page exposure, as measured using the high-profile Virginia Tech story, was 99.3% of ranked newspaper titles, with a cumulative print circulation of 99.8% of ranked newspapers. The latter figures indicate that the denominators of titles and counts of all ranked newspapers approximate to maximum coverage, and that US newspaper editors share common values when planning general high-profile front-page news.

This study also presents a new resource for quantifying coverage and identifying important media characteristics of front-page news—namely, *Newseum* in combination with the Top 200 rankings. Furthermore, using Top 200 rankings together with weighted circulation figures is more accurate for calculating news visibility than simply analyzing all newspaper titles, for two reasons. Firstly, unranked newspapers introduced bias—for example, unranked newspapers did not use staff writers in Story 2 and used news syndicates more commonly in Story 4 than in the control story (Table 2); inclusion of these titles thus affected the profiles of news sources (Table 1). Secondly, the data on newspaper titles alone misleadingly suggested that wire services and news syndicates are the most used sources of medical news.

When Top 200 newspapers and weighted circulation figures were considered, the patterns of known sources suggested the existence of different types of medical stories (Table 3). Story 1 seems most similar to Story 5 in coverage and news sources, preferring staff writers to syndicates or wires, while chi-square test results show that Stories 1 and 4 are not significantly different in profiles of sources. The topics of Stories 1, 4, and 5 (i.e. recall of medicine, stem cells, and college shootings) appear to be of both general and recent interest, as well as easily understood in terms of national importance or implications (e.g. regarding safety, ethics, and politics); such topics seem to be commonly assigned to staff writers. In comparison, Stories 2 and 3 (i.e. national cancer and MRSA rates, respectively) may be newsworthy for the reason of “routinely” updating the public on epidemiological data and trends, with such reports relying more on syndicates or wire services.

It is worrying that unauthored briefs were the most used form of front-page communication in three of the four medical stories in this study. Although most of them directed the reader to the full story on an inside page, the news source was undisclosed and the brevity (typically around 25 words) suggests insufficient or misleading front-page reporting for complex medical or health topics. The quality of such front-page elements warrants further research, especially as not all readers would refer to the full version. Incomplete or inaccurate reporting of medical news has previously been implicated in misinforming the public and even causing harm [17], [27], so front-page briefs regarding medical and health news should be carefully prepared.

Our findings show that three major newspapers (i.e. *Washington Post, Los Angeles Times, and New York Times,* which rank 6, 4, and 3, respectively, among the top 200 newspapers by circulation [*25*]) and the *Associated Press* wire service have an influential role in the reporting of front-page medical news in US newspapers. Together, these four sources accounted for more than 61% of the total coverage of the target medical stories. We speculate that media factors—including consideration of perceived high-quality content from reputable organizations and limited editorial budgets—may contribute to this situation. For example, the four sources employ dedicated medical writers (as indicated on their websites and occasionally on bylines). Additional factors—like the “technicality” of the news, reputation of a science journal or institution, and journal- or institution-produced press releases—may further affect how medical news is reported and distributed. The editorial processes of these four sources (e.g. editors' views of scientific research and use of medical writers) could be studied in future.

The four target medical stories, each with a cumulative daily circulation surpassing 23 million, overshadowed 5 to 17 low-profile medical stories, the most recurrent of which achieved a circulation of only 0.9 million. The appearance of different medical stories demonstrates the variable nature of how different newspapers prioritize medical news. Further investigation is needed to understand why such variability occurs, and how influential the *Washington Post*, *Los Angeles Times, New York Times* and *Associated Press* are in this decision, especially since these four dominant media organizations potentially bear a huge responsibility for public education and dissemination of medical and health information.

There are limitations to this study. Although we reviewed more than 300 newspaper front pages, a preliminary Internet search reveals a list of more than 1000 US newspapers, many of which are local publications. However, it would be impractical to assess every US newspaper and, if included in this study, many additional titles would not be in the Top 200 rankings by circulation. We acknowledge that not all Top 200 newspapers submit front pages to the *Newseum* repository, but capturing as many as 75% of ranked newspapers is as extensive as any research group has performed thus far. Many research groups use *LexisNexis* to capture newspaper content but this database provides a searchable list of only about 50 US newspaper titles. Because of the additional workload, we did not account for online newspaper versions or conduct a geographic survey or visibility audit (e.g. page position/area, word count, headline size, and use of graphics and color). Content analyses would be particularly useful to compare different news sources in their quality of coverage (e.g. accuracy, benefits, risks, expert opinions, and caveats); content (e.g. topic, technicality, tone, comprehensibility, and recommendations); and use and reporting of different levels of medical evidence and mature or preliminary data sources [28]. It would also be interesting to track how information from institution or journal press releases, newswires, and syndicates is treated by staff writers, as only one staff writer disclosed the use of other sources.

In conclusion, front-page coverage of medical news from different sources is more accurately revealed by analysis of circulation counts than of newspaper titles. Three national newspapers and the *Associated Press* may account for a large proportion of front-page coverage medical news and may exert considerable influence on media coverage of medical news in general. Journals wishing to widen knowledge of research news and organizations with important health announcements should target at least the four dominant media organizations identified in this study.

## References

[1] Phillips DP, Kanter EJ, Bednarczyk B, Tastad PL (1991). Importance of the lay press in the transmission of medical knowledge to the scientific community.. N Engl J Med.

[2] Nelkin D (1995). Selling Science: How the press covers science and technology..

[3] Wilkes MS (1997). The public dissemination of medical research: problems and solutions.. J Health Commun.

[4] Kiernan V (2003). Diffusion of news about research.. Science Communication.

[5] Angell M, Kassirer JP (1994). Clinical Research - What Should the Public Believe.. N Engl J Med.

[6] Nelkin D (1996). An uneasy relationship: the tensions between medicine and the media.. Lancet.

[7] Radford T (1996). Influence and power of the media.. Lancet.

[8] Campion EW (2004). Medical research and the news media.. N Engl J Med.

[9] Moynihan R (2003). Making medical journalism healthier.. Lancet.

[10] Marshall E (1998). Science and the media - The power of the front page of the New York Times.. Science.

[11] Danovaro-Holliday MC, Wood AL, LeBaron CW (2002). Rotavirus vaccine and the news media, 1987–2001.. JAMA.

[12] Woloshin S, Schwartz LM (2006). Media reporting on research presented at scientific meetings: more caution needed.. Medical Journal of Australia.

[13] PEJ (2008). The state of the news media 2008. Project for Excellence in Journalism.. http://www.journalism.org.

[14] Kennedy GE, Bero LA (1999). Print media coverage of research on passive smoking.. Tobacco Control.

[15] Schwartz LM, Woloshin S (2002). News media coverage of screening mammography for women in their 40s and tamoxifen for primary prevention of breast cancer.. JAMA.

[16] Kiernan V (2003). Embargoes and science news.. Journalism & Mass Communication Quarterly.

[17] Moynihan R, Bero L, Ross-Degnan D, Henry D, Lee K (2000). Coverage by the news media of the benefits and risks of medications.. N Engl J Med.

[18] Adelman RC, Verbrugge LM (2000). Death makes news: The social impact of disease on newspaper coverage.. Journal of Health and Social Behavior.

[19] CHPA (2007). Makers of OTC cough and cold medicines announce voluntary withdrawal of oral infant medicines. Consumer Healthcare Products Association.. http://www.chpa-info.org/ChpaPortal/PressRoom/NewsReleases/2007/10_11_07_CCMedicines.htm.

[20] Espey DK, Wu XC, Swan J, Wiggins C, Jim MA (2007). Annual report to the nation on the status of cancer, 1975–2004, featuring cancer in American Indians and Alaska natives.. Cancer.

[21] Klevens RM, Morrison MA, Nadle J, Petit S, Gershman K (2007). Invasive methicillin-resistant Staphylococcus aureus infections in the United States.. JAMA.

[22] Takahashi K, Tanabe K, Ohnuki M, Narita M, Ichisaka T (2007). Induction of pluripotent stem cells from adult human fibroblasts by defined factors.. Cell.

[23] Yu JY, Vodyanik MA, Smuga-Otto K, Antosiewicz-Bourget J, Frane JL (2007). Induced pluripotent stem cell lines derived from human somatic cells.. Science.

[24] Lee-St John J (2007). Top Ten News Stories of 2007. Time.. http://www.time.com/time/specials/2007/top10/article/0,30583,1686204_1690170_1691130,00.html.

[25] (2007). ABC Top 200 Newspapers by Largest Reported Circulation. Audit Bureau of Circulations.. http://www.accessabc.com/products/top200.htm.

[26] Ray S, Britschgi M, Herbert C, Takeda-Uchimura Y, Boxer A (2007). Classification and prediction of clinical Alzheimer's diagnosis based on plasma signaling proteins.. Nature Medicine.

[27] Schwitzer G (2008). How do US journalists cover treatments, tests, products, and procedures? An evaluation of 500 stories.. PLoS Medicine.

[28] Lai WYY, Lane T (2009). Characteristics of medical research news reported on front pages of newspapers.. PLoS ONE.

